# Atypical Presentation of *Haemophilus influenzae* Septic Arthritis: A Case Report

**DOI:** 10.5811/cpcem.2021.9.54043

**Published:** 2021-10-26

**Authors:** Andrew Steven Tadych, David Enrique Catano, April Lynn Brill

**Affiliations:** * Department of Clinical Education, Midwestern University – Chicago College of Osteopathic Medicine, Downers Grove, Illinois; † Department of Emergency Medicine, Midwestern University, Franciscan Health Olympia Fields, Olympia Fields, Illinois

**Keywords:** Haemophilus influenza, septic arthritis, HIV, synovial fluid white cell count, human immunodeficiency virus

## Abstract

**Introduction:**

Septic arthritis is a destructive form of acute arthritis that requires expeditious recognition. as delayed treatment yields significant morbidity and mortality.

**Case Report:**

A 40-year-old male presented to the emergency department with right elbow pain. Examination revealed tachycardia, swelling, redness, tenderness, and decreased range of motion of the right humeroulnar joint. Synovial fluid analysis was consistent with an inflammatory etiology, but blood and joint cultures ultimately revealed *Haemophilus influenzae.*

**Discussion:**

This case highlights the importance of trusting clinical findings over laboratory evidence in patients with suspected septic arthritis.

## INTRODUCTION

Septic arthritis (SA) is a key differential to consider in any case of acute monoarticular arthritis, as failure to initiate appropriate antibiotic therapy within 24–48 hours of onset can facilitate subchondral bone loss, permanent joint dysfunction, and lead to systemic spread of the infection.^1^ Historically, the likelihood of SA increases substantially with increasing synovial fluid (SF) white blood cell (WBC) count, in addition to other markers including serum leukocyte count, erythrocyte sedimentation rate, and C-reactive protein levels, which are often used to guide diagnosis.^2^ Conventional thinking suggests using a SF WBC count cutoff of 50,000 cells per cubic milliliter (cells/mm^3^) to guide diagnosis of SA.^3^ However, atypical cases of SA in patients with normal vitals, normal inflammatory markers, and inconclusive synovial fluid analyses have been documented.^4^

The most common pathogens isolated in cases of SA include *Staphylococcus aureus* and *Streptococcus pneumoniae*. Other less common pathogens include Gram-negative bacilli, mycobacteria, Gram-negative cocci, Gram-positive bacilli, and anaerobes.^1^ *Haemophilus influenzae* has been identified as a rare cause of SA, which had previously decreased in prevalence secondary to *H influenzae* serotype b (Hib) conjugate vaccine development in 1986.^5^ *H influenzae* is an encapsulated, pleomorphic Gram-negative rod with multiple capsular serotypes and commonly colonizes the human respiratory tract.^6^ While Hib is considered the most virulent serotype, other serotypes and non-typable *H influenzae* have been implicated in the development of invasive disease. Some invasive infections include meningitis, bacteremia, epiglottitis, SA, cellulitis, purulent pericarditis, endocarditis, and osteomyelitis.^7^ Therefore, the early identification and treatment of *H influenzae* SA is crucial in preventing significant morbidity and mortality.

The diagnosis of SA is made with a combination of clinical suspicion, positive blood cultures, and suggestive synovial fluid findings. Clinical presentation is variable, but often includes fever and malaise with localized pain, warmth, swelling, and decreased range of motion of the joint.^1^ Blood cultures are often drawn and can aid in the identification of infectious agents since SA is most often caused by hematogenous seeding of a joint.^8^ Important synovial fluid studies include Gram stain, leukocyte count, crystal analysis, and fluid culture. Traditionally, a SF WBC count of 50,000 cells/mm^3^ was used as the cutoff for diagnosing SA^3^; however, while increasing SF WBC count is associated with increased likelihood of SA, a low count is insufficient to rule out SA.^9^ Therefore, practitioners must rely on their clinical judgment when evaluating patients for SA in the face of inconsistent laboratory results.

## CASE REPORT

We present the case of a 40-year-old male with no significant past medical history, aside from a penicillin allergy, who presented to the emergency department (ED) with a chief complaint of severe right elbow pain, swelling, and limited passive and active range of motion for one day. He reported a prodromal flu-like syndrome of subjective fever, chills, and myalgias with associated right-arm soreness for two days prior to symptom onset. The patient stated that his right elbow started to swell and he progressively experienced increased pain over the following day. He attempted to use heating pads and ice without symptomatic relief. On further history, he reported recent unprotected sex with a new sexual partner in addition to right knee and hip pain without a history of trauma. At the time of his presentation, he denied any penile discharge, burning during urination, or pain in other joints.

On physical exam, the patient was in moderate distress due to pain. His right elbow was erythematous, edematous, warm, and tender to palpation. Passive and active range of motion were limited and worsened pain. Mild effusion was noted on palpation of the joint. He was initially afebrile with a temperature of 99.9°F, mildly tachycardic to 110 beats per minute, normotensive, and had 100% oxygen saturation. Labs were remarkable for an elevated erythrocyte sedimentation rate of 68 millimeters per hour (mm/hr) (reference range: 0–30 mm/hr), borderline leukocytosis of 10.7*10^3^ cells per milliliter (mL) (4.0–11.0*10^3^ cells/mL), mild hyponatremia, and mild hypokalemia. Complete blood count, creatinine kinase, and lactic acid were within normal limits. Blood cultures were obtained for Gram stain and culture.

Right elbow radiograph revealed joint effusion without evidence of fracture or soft tissue swelling (Image). Arthrocentesis from the right elbow was performed with 18 mL of aspirate. Synovial fluid analysis revealed cloudy fluid with small clots, a SF WBC count of 22,000 cells per cubic millimeter /(mm^3^) (reference range: 0–200 cells/mm^3^), 90% segmented neutrophils, and 66,500 red blood cells. Synovial fluid was cultured and sent for Gram staining. Intravenous (IV) ketorolac and morphine were given for pain control, and the patient was empirically started on IV clindamycin for suspected skin and soft tissue infection. While he was in the ED, there was suspicion for an inflammatory process of the joint with an overlying soft tissue infection. However, due to the risk of SA, he was admitted to the floor for further workup and evaluation.

On the floor, antibiotics were switched to aztreonam and vancomycin by the primary team due to suspicion of SA. Rapid human immunodeficiency virus (HIV)-1/2 antibody/antigen and gonorrhea-chlamydia tests were ordered. Orthopedic and infectious disease teams were consulted for further management and recommendations. Further laboratory testing revealed HIV 1/2 antibody positive, absolute cluster of differentiation (CD4) count of 82 cells/mm^3^ (reference range: 518–1,472 cells/mm^3^), CD4:CD8 of 0.2 (0.9–5.0), and positive blood cultures for *H. influenzae*. Upon blood culture results, the infectious disease team switched antibiotics to ceftriaxone and vancomycin to cover for *H influenzae* bacteremia. Due to the low CD4 count of 82 cells/mm^3^, trimethoprim-sulfamethoxazole was started for *Pneumocystis jirovecii* pneumonia (PJP) prophylaxis. The patient underwent right elbow arthrotomy, irrigation, and excisional debridement of the synovium and subcutaneous tissue. Postoperatively, he experienced intermittent fevers that responded to acetaminophen and improved over a few days.

**CPC-EM Capsule**

What do we already know about this clinical entity?

*Septic arthritis is a destructive form of acute arthritis that requires expeditious recognition as delayed treatment yields significant morbidity and mortality.*

What makes this presentation of disease reportable?

Haemophilus influenzae* septic arthritis is rare due to the available immunization. However, in this case the patient had an undiagnosed immunocompromised state.*

What is the major learning point?

*This case highlights the importance of trusting clinical findings as well as a thorough history over laboratory evidence in patients with suspected septic arthritis.*

How might this improve emergency medicine practice?

*Clinical presentations which are suspicious for septic arthritis must prompt careful diagnostic consideration, despite inconsistent laboratory findings.*

During his hospital course, the patient experienced polyarticular joint pain in his right knee and hip with swelling and difficulty weight bearing. This development was concerning for spread of *H influenzae* to the right hip and/or knee. Magnetic resonance imaging (MRI) of the right hip revealed trace effusion with edema within and surrounding the inferior segment of the visualized right iliopsoas muscle. This MRI finding was concerning for myositis secondary to *H influenzae* bacteremia. The patient then underwent aspiration of the right knee, which revealed no organisms and a SF WBC count of 3057 cells/mm^3^. Aspiration of the right hip also revealed an unremarkable SF analysis. Given systemic symptoms, echocardiogram was performed to rule out endocarditis and was negative for vegetations. As pain, swelling, and fever improved, a peripherally inserted central catheter (PICC) line was placed for outpatient IV antibiotics.

After eight days of in-patient evaluation and treatment, the patient’s clinical status and acute febrile illness had improved significantly. Per infectious disease final recommendations, the patient was discharged with a PICC line to receive IV ceftriaxone 2 grams daily for four weeks. He was also instructed to continue trimethoprim-sulfamethoxazole for PJP prophylaxis and to follow up with infectious disease as an outpatient to begin anti-retroviral therapy.

## DISCUSSION

This case demonstrates a rare presentation of *H influenzae* SA in an immunocompromised adult. Septic arthritis in a newly diagnosed human immunodeficiency virus (HIV)-positive adult can progress to a polyarthritic infection and further invasive disease. Therefore, early recognition and aggressive treatment combining surgical and medical modalities is often needed for optimal recovery. Unfortunately, as this case shows, there is no established guideline for ruling out SA. Given the presentation of severe pain, limited range of motion, erythema, warmth, and edema of the right elbow, the clinical suspicion for SA was initially high. This suspicion prompted an orthopedic evaluation and arthrocentesis. With a SF WBC count of 22,000 cells/mm^3^ our suspicion for inflammatory bursitis with an overlying skin and soft tissue infection increased while SA became less likely. While accepting this diagnosis may have led to delayed treatment, it would have been reasonable based on a SF WBC count diagnostic cut-off of 50,000 cells/mm^3^ for SA.

Prior studies have attempted to identify an appropriate cutoff for SF WBC count in the identification of SA*.* Margaretten et al published a systematic review analyzing the likelihood ratios (LR) for multiple different SF cutoff levels. They found a LR of 0.32 in adults with SF WBC count less than 25,000 vs SF WBC count greater than or equal to 25,000, which had a LR of 2.9.^9^ A newer review by Long et al found that nearly half of all patients with SA had a SF WBC count under 28,000 cells/mm^3^.^10^ While the authors suggest using clinical judgment over laboratory cutoffs, this data illustrates how difficult the diagnosis of SA can be.

Other studies have shown how immunodeficiency further increases diagnostic difficulty due to changes in SF findings.^11^,^12^ Notably, immunosuppression increases risk of infection while lowering SF WBC count, which complicates diagnosis. Zalavras et al identified consistently lower SF WBC count in HIV-positive patients with SA when compared to immunocompetent patients with average counts of 40,500 and 69,000 cells/mm^3^, respectively.^12^ Contrasted to a SF WBC count of 22,000 cells/mm^3^ in a newly diagnosed HIV-positive patient, we see that immunosuppression has a significant yet inconsistent effect on SF WBC count. Altogether, this can make diagnosis of SA particularly difficulty. Nevertheless, this case demonstrates the risks of ruling out serious diagnoses based on laboratory data. While the patient was initially worked up for complicated skin and soft tissue infection with an underlying inflammatory condition, it was the emergency physician’s insistence on admitting the patient that led to the diagnosis and treatment while preventing further complications.

## CONCLUSION

Any patient presenting with an acute onset of monoarticular joint pain, erythema, warmth, and restricted range of motion should be carefully evaluated for septic arthritis. In cases of contradictory lab findings, clinical judgment should rely more heavily on clinical findings in cases of SA due to a lack of established guidelines. Physicians must also consider immunocompromising comorbidities or potential immunocompromised states when analyzing synovial fluid results. Timely and accurate diagnosis of SA can decrease complications and improve patient outcomes. Therefore, clinical presentations that are suspicious for SA must prompt careful diagnostic consideration, despite inconsistent laboratory findings.

## Figures and Tables

**Image:**
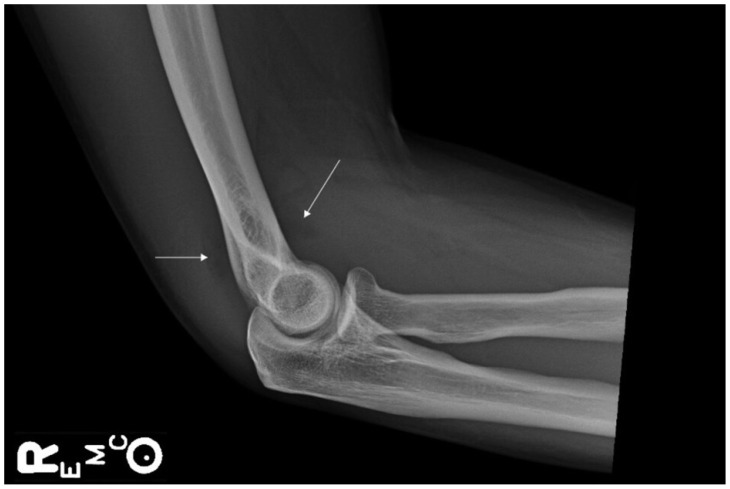
Lateral radiograph of right arm. Arrows identify displacement of the anterior and posterior fat pads consistent with a joint effusion.
